# Do depressive symptoms influence nonattendance at work? A semiparametric approach

**DOI:** 10.1007/s10754-025-09389-4

**Published:** 2025-03-04

**Authors:** Patricia Moreno-Mencia, Ana Fernández-Sainz, Juan M. Rodríguez-Póo

**Affiliations:** 1 Universidad Internacional de la Rioja, Av. La Paz, 136, Logrońo, Spain; 2 Universidad del País Vasco (UPV/EHU), E48015 Bilbao, Spain; 3 Departamento de Economía, Universidad de Cantabria, E39005 Santander, Spain

**Keywords:** Absenteeism, Depression, Semiparametric estimation, Control function estimators, Endogenous treatment effects, I15, J81, C21, C25.

## Abstract

Depression is a common disorder that impacts on individuals’ ability to perform daily activities, including those required for working. People with poor health tend to have problems needing medical care and therefore need time away from their work. This paper considers a structural model of labor absenteeism, considering the effect of depression. Our objective is to estimate the effects that depressive symptoms (among other factors) have on absenteeism while avoiding inconsistency in estimators due to sample selection and endogenous regressor. We are unwilling to impose strong assumptions, which are sometimes not required by theory, so our model is semiparametric. Based on microdata from the European Health Survey in Spain, our results indicate that depressive symptoms have a negative effect on working time and increase absenteeism. We conclude that depressed workers lose on average around 12 more days per year than non depressed ones. Levels of absenteeism are also estimated to be higher on average among obese people and among older people (the effect of age is positive). On the other hand, non-college education, being male and being self-employed are factors related to lower levels of absenteeism.

## Introduction

Depression is a very common disorder that impacts on individuals’ ability to perform life activities in general, and those required by work in particular (Fletcher, 2010; Berndt et al., 2002), and (Ettner, 1996). Ritchie and Roser (2018) estimate that 792 million people lived with a mental health disorder in 2017. This is slightly more than one in ten people globally ($10.7\%$). Moreover, the inconveniences caused by depression, which currently stand fourth on the global list of causes of death and disability, are expected to rise to second place, behind only heart disease, from 2020 on.

A review of the literature on depression shows that some depressive symptoms seem to differ in line with individual characteristics (age, gender, whether an individual suffers from an illness and other sociocultural factors). McNeil and Harsany (1989) show that depression in older populations is associated more with poor physical health than other factors. Moreover, the elderly tend to report emotional symptoms to their doctors to a lesser extent than younger adults do. Maes (2002) finds that among people aged $>55$ there is a greater prevalence of the usual depressive symptoms such as weight problems, a depressed state of mind, loss of interest, insomnia, anxiety, loss of self-esteem, psychotic symptoms and a slowing down in psycho-motor activity. Many studies have also focused on the causes of depression, with inheritance, personal circumstances, and co-existent illnesses being found to stand out (Santrok, 2001). Moussavi et al. (2007) concluded that depression is associated with a substantial decrease in health compared to the chronic diseases angina, arthritis, asthma, and diabetes. Duque-González (2009) estimates that mental illnesses are the second highest cause of indefinite and temporary sick leave from work, besides being the fourth highest reason for requiring informal care (that provided by non-professional carers, mostly family). Data from 2002 puts the total cost of mental illness in Spain at around 7.019 billion Euros, with direct medical costs accounting for 39.6% on that figure, i.e. around 7.3% of the total public health expenditure in that year. Depressed people are well known to feel unmotivated with jobs, and to go though long periods of inactivity or even give up their jobs for good. This illness is one of the most common reasons for seeking primary healthcare. It is also associated with the rate of chronic disability in individuals.

There is a large body of empirical research into the links between depression and labor participation and also the costs associated with it. In that sense, one concern is the issue of endogeneity. There are a few studies which use instrumental variables, e.g. Ettner (1996) uses the mental problems of parents as an instrument in patients’own mental disorders. Alexandre and French (2001) use variables associated with religion as an instrument for mental disorder, and show that depression entails a reduction of around $19\%$ in the probability of being in work. Martinez and Vall (2020) show that a deterioration in local labor market conditions is associated with a reduction in the consumption of anxiolytics. In labor economics, absenteeism is defined as non-attendance of employees for scheduled work (Frankel, 1921). For an individual, choosing whether to miss work is related to the costs and benefits of doing so, which often vary according to personal characteristics such as gender, age and health status. The repercussions of labor absenteeism in terms of productivity, financial expenses, and social impact are a topic of interest for the World Health Organization (WHO), which describes labor absences as an important, growing public health problem. It is also believed that this inability to work may cause economic losses to the tune of between $10\%$ and $20\%$ of the Gross Domestic Product (GDP) of a country (WHO, 2009). Labor absenteeism is thus a problem which affects not only individuals but also company managers and governments. According to the OECD (2018), the average period of paid absence from work due to illness in Spain in 2016 was 11.1 days (the equivalent figure was higher for Germany at 18.6 days but much lower for the United Kingdom at 2.2 days). Stress and associated problems due to anxiety and depression are the main causes of low happiness levels and are responsible for leaves of absence of more than 14 days, especially in sectors such as health and social work, education, and public administration according to the WHO (2017). Thus, the health consequences of depression are substantial and it is of great interest to examine them. Claxton et al. (1999) analyzes the effect of taking antidepressants on absenteeism among workers with depression. They find that absenteeism increases before workers start to take antidepressants and is reduced after treatment begins. There is a large body of literature focused on the impact of the health of individuals on continuity in the labor market (see Mashonganyka (2004) and Garcia-Gomez (2011) among others). There are also studies which analyze the negative health and economic aspects of absenteeism (Galle et al., 2019). Other studies examine the impact of mental disorders on the labor supply. Depression also has an adverse effect on employment that may result in presenteeism, absenteeism, and job loss. In most cases people with bad health, and especially with depression, have problems that require medical care and therefore frequently need time away from their work. The European Survey of Health in Spain 2014 found the 12-month prevalence of depression among the working population of Spain to be $4\%$ overall ($6\%$ for women and $2\%$ for men). There are several studies that link depression with labor market outcomes, but research into the intensity of participation is less common. The labor supply is associated with health outcomes (Deaton & Paxson, 1998; Currie, 2009) and in that sense the hypothesis explored here is that there is a negative link, i.e. worse health means fewer hours worked.

Johnston et al. (2019) uses a univariate linear regression and confirm a negative linear relationship between severity of depression and presenteeism which remains significant after age, gender, industry, and work position are controlled for. Hendriks et al. (2015) concluded that depressive syndromes and symptoms have more impact on future work disability and absenteeism than anxiety, which implies that prevention of depression is of major importance. Kessler and Frank (1997) show that the average prevalence of work-loss days for psychiatric reasons (6 days per month per 100 workers) and work-cutback days (31 days per month per 100 workers), in comparison, do not differ significantly across occupations. Along the same lines, Uribe et al. (2017) use an Ordinary Least Squares Estimation to examine hours of absenteeism in the last month in Colombia, and find that the average number of hours per month lost to absenteeism is 43 (more or less 5 days per month). Wada et al. (2013) work out the wage losses due to the absenteeism. They show that individual wage losses are higher among those with depression, anxiety, and other disorders. Rost et al. (2004) analyze whether an intervention to improve primary care depression management is associated with greater productivity at work and less absenteeism over 2 years. They conclude that improving the quality of primary care has positive consequences for both, productivity and absenteeism.

In short, an analysis of depression from an economic point of view is of great interest because of the substantial increase in the number of people with depression in the past few decades and the effects that this disorder has on the labor market and on society as a whole. According to King et al. (2008), the Spanish are the Europeans who suffer most frequently from depression and anxiety; by contrast the people of the Netherlands and Slovenia are the least likely to suffer mental health problems.

The main difficulty in analyzing problems of this kind is that the standard econometric model may be subject to sample selection (not all working-age individuals are in work), so it is necessary to correct the bias as otherwise estimates will be biased and inconsistent (Greene, 2003). Moreover, one of the covariates considered (whether depressive symptoms are suffered or not) is endogenous to the model (unobservable factors affecting absenteeism are correlated with depression). Examples of this problem in a fully parametric context include Manning et al. (1984) in health economics for managing health care studies and Heckman (1974) in labor economics for studying women’s labor supply. The main issue with the estimation techniques used in these empirical applications is that the assumptions rely on a fully parametric specification. It is well known that there are at least two arguments against using parametric techniques: First-stage Probit maximum likelihood estimators, under possibly non-normal errors, are inconsistent and then the Inverse Mills‘ Ratio (IMR) is misspecified, so second step estimators will be inconsistent. Certain semi- and non-parametric estimation procedures in the context of sample selection models have become popular in theoretical literature (Ahn & Powell, 1993) and (Das et al., 2003) among others) but they have seldom been applied to heath economics. Due to these two important problems of estimator inconsistency, we propose an alternative two-step estimation procedure. In the first step we specify functions which are non-parametrically estimated, applying the proposal in Das et al. (2003). In the second stage the parameters of the initial model are calculated using the weighted data from the first stage. The main advantage of this approach is that it is not necessary to know the conditional distribution of the disturbance terms given the explanatory variables, so the estimators are robust to any misspecification in the error distribution. The idea for identifying and estimating the model is to include a control function of the estimated correction terms obtained in the first step in the main model as an additional regressor (second step).

Our objective is to estimate the effect of depression in a structural model of labor absenteeism, taking into account both sample selection bias and endogeneity. In short, we estimate an absenteeism model for workers with depressive symptoms that takes into account the two econometric problems that arise, i.e. sample selection bias (since not all the active population is in work) and the endogeneity of one of the covariates (having depressive symptoms, which is simultaneously correlated with other unobservable factors affecting absenteeism, such as general self-perceived health status). The main contribution of this study is that within the class of pairwise difference estimators we propose a new weighting function that emerges when an endogenous switching Type II- Tobit Model is considered.

The paper is organized as follows; Section 2 describes the econometric model and how to implement the estimation techniques required. Section 3 analyzes labor absenteeism among sufferers from depression and shows the results. Section 4 presents discussions and Section 5 concludes.

## Materials & methods

### Data

The European Health Survey in Spain (EHSS) is conducted on individuals aged over 15 who reside in family households (INE, 2014). Its main objective is to collect information on health status, use of health services and factors which affect health. This dataset is comparable for all European countries and is usually collected every five years, with the most recent one available here dating from 2014. The survey enables micro-data to be used on two possible levels of analysis: Spanish households and the individuals who lived in them. The analysis reported here uses the individual level, so there is information on socioeconomic characteristics and chronic illnesses affecting individuals. The sample size comprises around 23, 000 households distributed across 2, 500 census sections with questions answered by 22, 842 individuals. In each household one adult (aged 15 or over) is selected to answer the questionnaire. The geographical scope covers the whole of Spain, and is thus representative of the Spanish population. The information is collected via computer-assisted personal interviews (CAPI), supplemented when necessary and in exceptional cases by means of telephone interviews.

### Statistical methods

In the analysis proposed our interest is in labor absenteeism, so only individuals currently in work can provide information about how many days’ work they have missed. This is a possible source of bias due to sample selection. Moreover, if there are unobservable factors associated with labor absenteeism such as discrimination at work which may also affect the onset of depressive symtoms, then an endogeneity problem also arises. We set out to analyze this by accounting for selection bias using a traditional sample selection model (Heckman, 1979) and considering the following structural equation model:

1 $$\begin{aligned} y_i = \left\{ \begin{array}{ll} y^*_i & \quad {\text {f}or} \quad p_i = 1, \\ 0 & \quad {\text {o}therwise}. \end{array} \right. \end{aligned}$$

2 $$\begin{aligned} p_i = 1\left( p^*_i > 0\right) , \end{aligned}$$

where

3 $$\begin{aligned} y^*_i= &  w^{\top }_{1i}\beta _1 + \lambda _1d_{i} - \eta _{1i}, \end{aligned}$$

4 $$\begin{aligned} p^*_i= &  g_2( w^{\top }_{2i}\beta _2)- \eta _{2i}, \end{aligned}$$

$p_i$ is one if individual *i* has a job and zero otherwise; $y_{i}$ is the potential outcome (days of nonattendance) for individual *i*; $d_i$ is the binary endogenous regressor, which is one if individual *i* has depressive symptoms and zero otherwise; $w_{1i}$ is a random vector of observed covariates; $w_{2i}$ includes all variables in $w_{1i}$ and an additional regressor for the purpose of identification, which is the endogenous binary regressor $d_i$. Depressive symptoms are explained through the following mechanism:

5 $$\begin{aligned} d_i = 1\left( g_3(w^{\top }_{i}\beta _3)-\eta _{3i}> 0\right) , \end{aligned}$$

$w_{i}$ includes $w_{1i}$ and an additional regressor for the purpose of identification. Thus, $\beta _1$ and $\lambda _1$ are the set of parameters of interest and $g_2(\cdot )$ and $g_3(\cdot )$ are unknown nonparametric functions that also need to be estimated.^1^ In the European Health Survey, the mental health submodule aims to evaluate the prevalence of depression according to the criteria of the Diagnostic and Statistical Manual of Mental Disorders. To achieve this, the survey employs the Patient Health Questionnaire (PHQ-8) in the adult questionnaire. The PHQ-8 is a very used validated tool consisting of eight questions that ask respondents to rate the frequency of specific symptoms (e.g., feelings of sadness, loss of interest in activities, changes in appetite, sleep disturbances) over the past two weeks. Each question is scored from 0 (not at all) to 3 (nearly every day), with the total score indicating the severity of depression.For our analysis, we transformed these ordinal categories into a binary variable to indicate the presence or absence of significant depressive symptoms.

In this estimation context, it is possible to disaggregate the above model as is shown in the Appendix.

The problems faced in this model are the following: (i) $\eta _{1i}$ may be correlated with $d_i$ so methods such as Ordinary Least Squares provide biased, inconsistent estimates; and (ii) $d_i$ is included as a component of $w_{2i}$, so the (dummy) endogenous regressor is also present in the selection process.

Taking the single index methods into consideration, several approaches can be found in recent literature for dealing with these issues. The main advantage of this methodology is that there is no need to know the conditional distribution of the disturbance terms given the regressors, so the estimators are robust to possible misspecification in the distribution of disturbances. Powell (1987) proposes estimating the unknown parameters included in the index function with a semiparametric estimation procedure (Nadaraya, 1965, Klein and Spady 1993 or Manski, 1975). We therefore base our approach on flexible methods of this kind for estimating first step propensity scores.

It must be remembered that our objective is to estimate the parameter set $(\beta _1, \ \lambda _1)$ considering the existence of unknown functions $g_2(\cdot )$ and $g_3(\cdot )$, without assuming any pre-specified distribution for errors.

### Estimation techniques

Following the approach of Heckman (1979) and Vella (1993) the parameters $\beta _1$ and $\lambda _1$ can be estimated through a two-step procedure. The idea here is to propose corrections terms for the problems of selectivity and endogeneity.

In this procedure^2^ one of the parameters of interest, $\lambda _1$, is not identified because of taking pairwise differences. In this case, this is an important problem since $\lambda _1$ is the parameter to be estimated.

The method proposed has two-steps:

- In the first step, following the estimation process proposed by the regression of Klein and Spady, $\hat{p}_{2i}^0,\hat{p}_{2i}^1$ and $\hat{p}_{3i}$ are obtained to estimate the correction terms by chosing a nonparametric model. Based on these probabilities, the parameters of the equation of interest ($\hat{\beta _1}$) are calculated with a kernel regression estimator which chooses weights so that observations whose propensity scores are close together receive greater weight.
- In the second step, taking into account that in the procedure the parameter to be estimated, $\lambda _1$, is not identified because differences have being taken, matching techniques are used to recover it.

## Results

### Summary statistics

Considering that our main interest is in the labor market, we restrict our sample to the population aged between 16 and 65 and select the active population (individuals in and out of work). The dependent variable (*y*) is the number of days’work that an individual has missed in the last year due to health problems (Labor Absenteeism). The selection variable (*p*) takes a value of one if the individual is in work and zero otherwise and the binary endogenous regressor (*d*) is the onset of depressive symptoms. That is, $d_i =1$ if the *i*-th individual suffers any depressive symptom and 0 otherwise. The covariates included in the model as controls are obesity, gender, self-employment, education level and age. The participation equation includes the aforementioned explanatory variables plus civil status. From a theoretical point of view, it is reasonable to think that being single is associated with the participation decision. Once the control variables are considered, we belive that this variable is uncorrelated with other omitted factors that affect absenteeism. The reduced equation on suffering depressive symptoms includes not just the said controls but also a variable denoting low income.^3^ This variable is directly related to depression (having a low income probably increases the likelihood of suffering depressive symptoms). When the control variables are considered this variable is probably uncorrelated with other omitted factors that affect labor absenteeism.

**Table 1:** Summary statistics: Mean and standard deviation (in brackets) Source: Own work based on the European Survey of Health in Spain, 2014

Variables	Definition	Whole sample	With Depressive symptoms	Without Depressive symptoms	Workers	Workers with depressive symptoms	Workers without depressive symptoms
Absenteeism	In days	–	–	–	6.09 (37.68)	14.60 (46.23)	5.27 (25.05)
Age	In years	43.49 (10.95)	47.15 (9.70)	43.09 (11.01)	43.36 (10.82)	47.12 (9.86)	43 (10.84)
Elementary	1, if elementary schooling	0.113 (0.317)	0.119 (0.325)	0.113 (0.317)	0.058 (0.23)	0.071 (0.25)	0.057 (0.23)
High School	1, if High School	0.245 (0.430)	0.249 (0.432)	0.244 (0.430)	0.273 (0.44)	0.317 (0.46)	0.268 (0.44)
College	1, if College	0.142 (0.349)	0.123 (0.329)	0.144 (0.351)	0.213 (0.40)	0.209 (0.40)	0.213 (0.40)
Male	1, if male	0.526 (0.499)	0.382 (0.486)	0.542 (0.498)	0.534 (0.49)	0.383 (0.48)	0.549 (0.49)
Single	1, if single	0.272(0.445)	0.245 (0.430)	0.275 (0.445)	0.254 (0.435)	0.241 (0.428)	0.255 (0.436)
Low Income	1, if income $<970$€	0.122 (0.328)	0.096 (0.296)	0.125 (0.331)	0.056 (0.230)	0.024 (0.428)	0.059 (0.235)
Depressive	1, if Depressive sympt	0.099 (0.29)	–	–	0.087 (0.28)	–	–
Obesity	1, if obese	0.122 (0.32)	0.142 (0.34)	0.119 (0.32)	0.130 (0.33)	0.174 (0.37)	0.126 (0.33)
Employed	1, if employed	0.358 (0.47)	0.317 (0.46)	0.362 (0.48)	–	–	–
Self-Employed	1, if self-employed	0.045 (0.21)	0.028 (0.16)	0.047 (0.21)	0.085 (0.27)	0.060 (0.24)	0.087 (0.28)
Public employee	1, if public employee	0.031 (0.17)	0.026 (0.16)	0.031 (0.17)	0.062 (0.24)	0.059 (0.23)	0.063 (0.24)
N	Sample size	5327	526	4801	1908	167	1741

As can be seen in Table 1, $8.7\%$ of workers had symptoms of depression. The number of days of absence (14.6) among workers with depression was nearly three times that for workers without depressive symptoms (5.27). $14.2\%$ of obese individuals were found to be depressed and the percentage of men with depression ($38.2\%$) was much lower than for women ($61.8\%$). A look at education levels reveals that the highest percentage of people with depression was found among those with High School education (24.9%). $35.8\%$ of the overall active population were in work (1908 out of 5327), but among those suffering depressive symptoms the figure was only $31.7\%$ (167 out of 526). People miss work for a variety of reasons. According to the WHO (2017), the main causes of absenteeism include stress and tiredness; childcare and elder-care, feeling bullied at work, depression and other illnesses and injuries.^4^

This paper focuses on just one of those causes: depression. The prevalence of depression and anxiety is set to increase in the future and a report by the World Health Organization (2017) predicts a growing role for depression among health problems for the entire world. It is estimated that nearly three million people in Spain suffered from depression ($5.2\%$ of the population) in 2015. Depression was also the second leading cause of sick leave in the country, where estimated spending on it totals 23 billion Euros per year. Depression is an increasingly common condition in Spain, where the latest figures suggest that between three and six million people suffer depressive symptoms (see WHO (2017) for more details).

**Table 2:** Absence from work (days) due to health problems Source: Own work based on the European Survey of Health in Spain, 2014

Age	Female	Male	Workers
16 - 24	0.77	6.84	4.07
25 -34	6.03	3.76	4.81
35 - 44	10.95	4.09	7.02
45 - 54	5.07	4.03	4.54
55 and over	11.49	7.08	9.23
Total (16 - 65)	7.79	4.62	6.09
Total (16 - 65) with depression	16.45	11.61	14.60
Total (16 - 65) without depression	6.65	4.15	5.27
N	888	1020	1908

Table 2 shows absences from work broken down by age and gender in the sample of individuals in work. As can be observed, there may be differences in behavior between men and women. According to this data, women on average miss more working days than men in all but the youngest age group. The age group with most days of absence is that of the over 55s, where the difference between men and women is about 5 days. A look at the sample of workers aged from 16 to 65 reveals that it is women with depression who miss most work, at almost 17 days. The difference between men and women suggests that it may be better to treat each group separately. However, their behaviors need to be examined to check whether there are different responses from one to the other. To determine whether it is necessary to divide the sample we use a Chow test, which is a formal test developed to test parametric stability between groups.^5^ The conclusion drawn is that there are no statistical differences between men and women so it is not necessary to separate the analysis by gender, though this should be taken into account as a control covariate.

### Model results

The relationship between depression and labor supply decisions is of longstanding interest in labor and health economics but it must be kept in mind that the variable of interest is usually censored and is also subject to sample selection (not all working age individuals are in work). To check the suitability of our estimator we propose an econometric analysis of labor absenteeism in Spain, comparing the results with those obtained using more standard methods (Ordinary Least Square, Two-Step Sample selection model, and Propensity Score Matching).

**Fig. 1:**
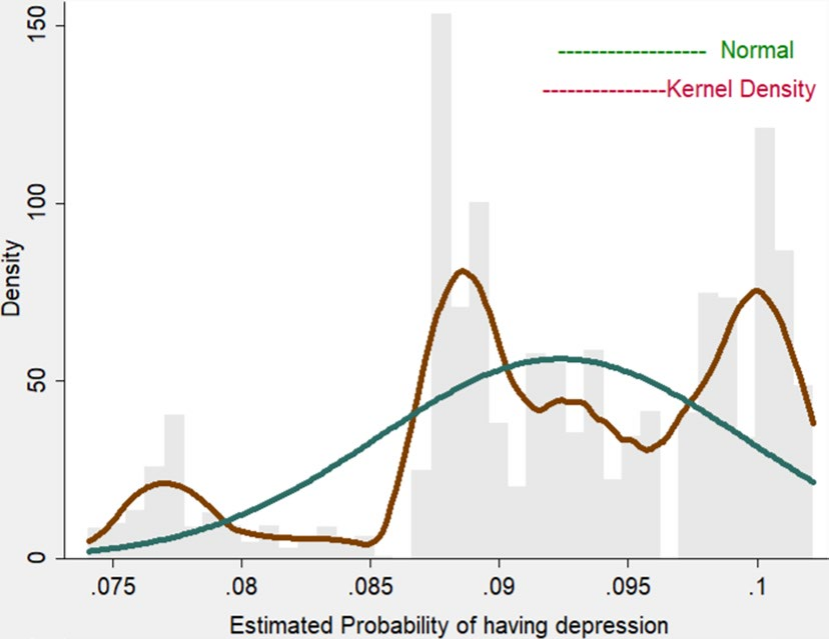
Predicted probability of depressive symptoms. Source: Own work based on the European Survey of Health in Spain, 2014

**Fig. 2:**
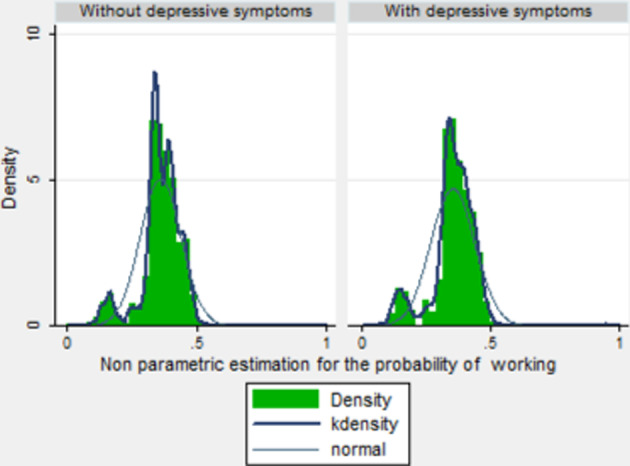
Predicted probability of working. Source: Own work based on the European Survey of Health in Spain, 2014

**Fig. 3:**
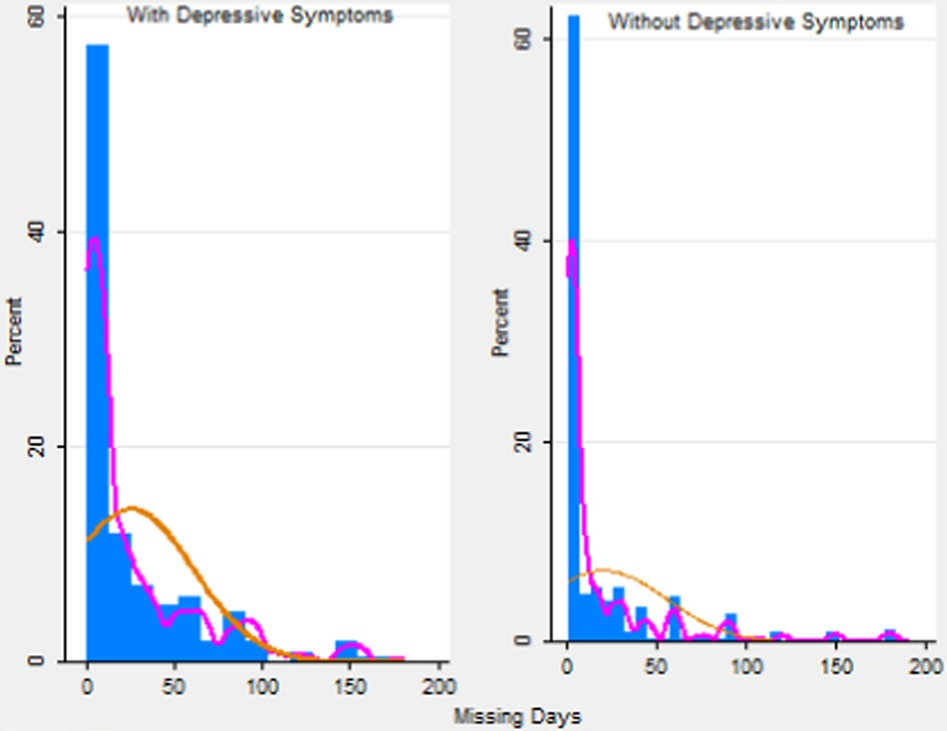
Days’Work Missed. Source: Own work based on the European Survey of Health in Spain, 2014

This section presents the results obtained.^6^ As indicated in the Statistical Methods subsection, the model is estimated in two steps:

- In the first step, a nonparametric model is chosen to estimate the correction terms, following the estimation process proposed by Klein and Spady regression, which gives $\hat{p}_{2i}^0,\hat{p}_{2i}^1$ and $\hat{p}_{3i}$. The kernel function used in this approach is the "biweight kernel". It is thus necessary to choose a bandwidth. The value chosen for the bandwidth is $h = 0.2$. It is usual to choose a fixed bandwidth, and given that the probabilities are between 0 and 1 either 0.2 or 0.5 would be an appropriate choice. The final choice is confirmed with visual interpretation (plot with numerous span values) and Cross Validation (CV). Figures 1 and 2 show the probability of suffering depression (for individuals not in work and those in work) and the density of the predicted probability of being in work (with and without depression). It can be observed that if the functional form is left unrestricted it seems to fit the model much better than if normal distribution is just assumed in both cases. The highest probability of being in work is about $43\%$ while the normal distribution shows that the most probable figure is $34\%$. As can be seen, there are higher figures for part of the population of around $43\%$ in the population without depressive symptoms. On the other hand, there is more accumulation around the lowest probabilities such as the $10\%$ probability of being in work for people with depressive symptoms. Additionally, the normal assumption overestimates the average probability of having depression. Figure 3 shows the percentages associated with different numbers of missing days (only for the population who missed at least 1 day) under parametric and semiparametric estimation procedures. As can be seen, the semiparametric estimation predict fewer days missed, on average, than the normal density and seems to fit the data better. It can also be seen that over $62\%$ of individuals without depressive symptoms miss only one day while for those with depressive symptoms the figure is about $58\%$. It is significant that the percentages associated with missing more days (i.e. 50 or 100 days) are clearly higher for those with depressive symptoms.
- The second step entails estimating the parameters of interest in the absenteeism equation (Table 3-column 4). Our results confirm that all the variables included are statistically significant. In particular, the categories of non-college educated men (with only elementary and high-school studies) and self-employed men have a negative impact on the expected number of working days missed, i.e. they miss fewer days’ work. By contrast, depressive symptoms and obesity are positively related with absenteeism. Scott and McClellan (1990) state that although women are expected to miss more days at work, their actual absences are not significantly different. Age has a positive effect and people with only elementary schooling are far more negatively related to absences. This may be explained by the fact that they have more temporary jobs or less income at home and are thus more afraid of losing their jobs (Tompa et al., 2008). The effect of depression on labor absenteeism (Table 3) thus means that a person with depression is expected to miss around 12 more days’ work than one with similar characteristics but without depression. Moreover, this effect is statistically significant. As mentioned, in the procedure used the parameter of interest ($\lambda _1$) is lost. To recover it, we use a Propensity Score Matching approach. Thus, Table 3 (column 3) shows the ATE (the difference in the average results between the units assigned to the treatment (depressed workers) and those assigned to the control group (not depressed)^7^. The ATE parameter is that for the variable *Depressive*.

**Table 3:** Estimation of the absenteeism equation Source: Own work based on the European Survey of Health in Spain, 2014

Absenteeism	OLS (Without Corrections)	Heckman-2-Steps (for sample selection)	PS-Matching (for endogenity)	Our Approach (Both corrections)
Obese	3.194*** (1.224)	2.917* (1.911)	0.110* (0.07)	2.293*** (1.195)
Male	$-$1.462* (0.805)	$-$2.905 (1.290)	$-$0.327*** (0.05)	$-$0.949** (0.790)
Self-Employed	$-$1.787 (1.948)	$-$0.865 (2.334)	$-$0.271* (0.134)	$-$2.614** (1.895)
Primary	$-$1.384 (1.283)	$-$0.264 (4.074)	0.057* (0.076)	$-$3.489*** (1.122)
High School	$-$1.475 (0.959)	$-$1.447 (1.502)	0.034* (0.601)	$-$1.563*** (0.778)
Age	0.120* (0.036)	0.047*** (0.059)	0.017*** (0.002)	0.222*** (0.036)
Depressive Symptoms	11.203*** (1.351)	8.572*** (2.253)	10.024*** (3.238)	11.962*** (2.406)
IMR	–	3.267*** (1.470)	–	–
N	1908	1908	1908	1908

Signif. codes: 0.01 ’***’ 0.05 ’**’ 0.1 ’*’

Finally, purely for the sake of comparison, Table 3 includes estimates obtained using other standard approaches, even though, as explained above, most of these approaches (e.g. OLS (column 1)) are not suitable for our problem. Our results can be seen to be quite close to those obtained with the Heckman sample selection model (column 2), though unfortunately the latter is inconsistent under endogenous regressors because the Inverse Mills ratio (IMR) is statistically significant, which means that there is a sample selection problem and OLS is not a suitable method. To gauge the robustness of our estimates, we run the Hausman-Wu test of exogeneity on the *Depressive* variable. The null hypothesis of exogeneity is rejected, so estimating this model without correcting for endogeneity is not suitable. The results obtained with a Propensity Score Matching approach can be seen in column 3, but in this case the selection bias due to labor market participation is not considered. The fact that the *Depressive* variable is significant means that the endogeneity problem is present in the estimation. The standard errors need to be constructed using bootstrapping. In this case, the standard errors are somewhat smaller than in the other methods.

In summary it can be concluded that (i) on average, depressed workers miss around 12 more days than non depressed ones; (ii) the effects of the control variables are statistically significant; and (iii) OLS, Propensity Score Matching and Heckman’s procedure underestimate the effect of depression.

## Discussion

Depression is an illness which has been increasing significantly in recent years (WHO, 2017). In this paper we conclude that it has a negative effect on labor market decisions. People suffering from depression have a low participation ratio and prefer not to work or to work fewer hours, which may increase absenteeism. There is therefore a quantifiable cost of depression, both for the individual in wage losses and for society in resulting medical and social costs.

Given these findings, managing depression correctly is clary important. Thus, it could be useful to adopt new policies and procedures to reduce risks and negative effects of suffering depressive symptoms. Efforts are currently being made to reduce labor absenteeism, and in that regard some companies seek to provide their employees with incentives to go to work (Martimo, 2006). Others focus on concern for improving employee health in aspects such as mental health or environment-related health. Finally, workers have to act responsibly with their jobs. Some absences from work are evidently inevitable and quite common but there is sometimes abuse related to a lack of morale (Lamichhane et al., 2018). Missed work days have a serious financial effect on firms, so implementing strategies to reduce absenteeism and manage it as well as possible may be a good course of action.

This paper makes several contributions to the literature on impacts on labor supply decisions: First, it uses microdata from the European Health Survey for 2014, one of the most comprehensive databases related to health and living conditions. Second, the presence of a dummy endogenous regressor in both the structural model and the selection equation distinguishes this analysis from those usually conducted, which account for continuous endogenous regressors only in the main equation. Finally, we specify the control function used to correct those problems as non parametric in order to avoid imposing strong traditional assumptions.

Although depressive symptoms are the variable of main interest, other important findings also emerge. Obese people are estimated to have higher levels of absenteeism on average. By contrast, lower education levels are related to less absenteeism, as is being self-employed. The effect of age is also positive, i.e. older people tend to miss more days.

This study analyzes for the first time, the relationship between depressive symptoms and absenteeism considering the sample selection and endogenity problems which are present in the model. Moreover, unlike earlier studies, we leave the control function unrestricted, which makes our model highly flexible.

As expected, we find a significant association between depression and workplace absenteeism. Similar results are obtained by Bouwmans et al. (2014), who find that more than half of working populations with depression, anxiety or both, suffer from long-term absenteeism (more than 2 weeks absent from work).

Our results provide very valuable additional information over and above the mere fact of the disorder itself, which should be taken into account in studying its economic cost in terms of absenteeism. In the specific context considered here, we estimate several methods for assessing the impacts of various factors to account for the differences in work days missed with or without depression.

Several important implications emerge from the paper. First of all, our findings indicate that depressive symptoms have a significant impact on absenteeism. Thus, to prevent long-term absenteeism more attention needs to be paid to the working environment and to early recognition and early attention so that people can access treatment as soon as possible, costs arising from productivity losses can be avoided and long-term absenteeism can be reduced. This has led governments to create economic and social policies to prevent depression or help sufferers to learn to live with it. Policies which can decrease rates of absenteeism and reduce the costs associated with it are also needed. As stated above, prevention and efficient treatment could have significant benefits not only in economic terms but also in quality of life. It can be concluded from this analysis that economic and social policies aimed at preventing illnesses and helping people live healthy lives should be implemented.

## Conclusions

This study estimates the incidence of suffering depressive symptoms in a labor absenteeism model. The increasing number of workers who miss days at work due to mental health problems has become a problem in our society. To solve it, various measures can been considered. Firstly, appropriate diagnosis and correct medical treatment are needed. Secondly, firms need to foster a healthy environment at work. Taking this into account, we find that absenteeism is costly for both employees and employers so it is a field that is relevant and that needs to be studied.

This paper applies a two-step estimation method in a cross-sectional model with a binary endogenous variable and sample selection. This estimator can be considered as an extension of Heckman’s two-step estimator, allowing for two different problems which are faced simultaneously (sample selection and endogeneity). Moreover, the paper does not impose the normality assumption. To our knowledge, this is the first time that this type of specification has been applied to estimate days of absence from work.

To apply the econometric method, we derive correction terms for the selection and endogeneity problems, enabling us to obtain consistent estimates for the structural equation. We present the assessment of the impact of depressive symptoms on expected work days missed as an empirical application. The estimated effect indicates that people who have depressive symptoms miss around 12 more days’work than those who do not. This estimate is significant and positive as expected, because less healthy people may feel unmotivated at work, may have less energy and may devote less time to pursuing promotion.

In the study of differences in work days missed labor participation decisions are affected by the onset of depression. We apply various methods and arrive at robust conclusions as to the effect of depression as a specific illness, on labor decisions.

As future lines of research it could be interesting to consider outcome variables other than absenteeism, such as working hours. Another good extension could be to compare the results for Spain with those for other OECD countries. We also suggest investigating the extent to which mental disorders reduce workers productivity and the causes for this. Another important point is understanding the disjunction between absenteeism and presenteeism for people with mental problems.

Several limitations of this study should also be noted. First, the results do not consider other important mental disorders such as anxiety, obsessive compulsive disorder, post traumatic stress and other specific phobias. Secondly, abseenteism is self-reported, so it could be subject to measurement errors and bias. In this sense it would be preferable to have administrative data but such data is not always available. However, some studies have shown that self-reported data on absenteeism is closely correlated with employer payroll records (Kessler, 2003). Moreover, self-reporting data can introduce bias, but recognizing and addressing this potential limitation through careful study design and robust analytical methods can help mitigate its impact on research findings.

## Appendix

The model made from equations (1)-(5) can be disaggregated as follows:

$$\begin{aligned} y^*_i= &  d_i y^*_{1i} + (1-d_i) y^*_{0i}, \\ p^*_i= &  d_i p^*_{1i} + (1-d_i) p^*_{0i} \end{aligned}$$

where developing the switch results in:

$$\begin{aligned} y^*_{1i}= &  w^{\top }_i\beta _1 + \lambda _1 + \eta _{1i}^1, \\ y^*_{0i}= &  w^{\top }_i\beta _1 + \eta _{1i}^0, \\ p^*_{1i}= &  g_2(w^{\top }_{2i}\beta _2|d_i^*>0) + \eta _{2i}^1, \\ p^*_{0i}= &  g_2(w^{\top }_{2i}\beta _2|d_i^*<0)+ \eta _{2i}^0 \end{aligned}$$

$p^*_{1i}$ denotes the probability of working for an individual suffering from depressive symptoms while $p^*_{0i}$ denotes the probability of working for an individual without depressive symptoms.

$y_{i}$ is the potential outcome (days of nonattendance) for individual *i*; if he/she is affected by the disorder the potential outcome is denoted by $y_{1i}$ and if not by $y_{0i}$. Note that the difference $y_{1i}-y_{0i}$ is the effect on the expected outcome for person *i* of having depression.

Substituting the desegregated models in equations (3)-(4) gives the following:

6 $$\begin{aligned} y^*_i= &  w^{\top }_{1i}\beta _1 + \lambda _1d_{i} + d_i\eta _{1i}^1 + (1-d_i) \eta _{1i}^0, \end{aligned}$$

7 $$\begin{aligned} \nonumber p^*_{i}= &  g_2(w^{\top }_{2i}\beta _2|d_i^*<0) + d_i\left( g_2(w^{\top }_{2i}\beta _2|d_i^*>0)- g_2(w^{\top }_{2i}\beta _2|d_i^*<0)\right) \\+ &  d_i\eta _{2i}^1 + (1-d_i)\eta _{2i}^0. \end{aligned}$$

If conditional expectations are taken then:

8 $$\begin{aligned} E\left( y_{i}|w_i,d_{i}, p_{i}^*>0\right)= &  w^{\top }_{1i}\beta _1 + d_i\lambda _1 + d_i E\left( \eta _{1i}^1|w_{1i},p^*_{i}>0,d^*_{i}>0\right) \nonumber \\ &  + \left( 1-d_i\right) E\left( \eta _{1i}^0|w_{1i},p^*_{i} > 0,d^*_{i}\le 0\right) . \end{aligned}$$

So, given that the aim here is to estimate $\beta _1$ and $\lambda _1$ consistently, $E\left( \eta _{1i}^{1}|w_{1i},p^*_{i}>0,d^*_{i}>0\right)$ and $E\left( \eta _{1i}^{0}|w_{1i},p^*_{i}>0, d^*_{i}\le 0\right)$ must be computed. Following Das et al. (2003) those unknown expressions may be written as, $E\left( \eta _{1i}^{1}|w_i,p_i^*>0,d_i^*>0\right) = \mu _1\left( p_{2i}^1,p_{3i}\right)$ and $E\left( \eta _{1i}^0|w_i,p^*_i>0,d^*_i<0\right) = \mu _0\left( p_{2i}^0,p_{3i}\right)$.

The propensity scores are estimated as follows:

9 $$\begin{aligned} p_{3i}= &  E\left( d_i\left| w_i\right. \right) ; \end{aligned}$$

10 $$\begin{aligned} p_{2i}^1= &  E\left( p_i\left| w_{2i},d_i=1\right. \right) = E\left( p_i\left| w_{2i}^1\right. \right) \end{aligned}$$

11 $$\begin{aligned} p_{2i}^0= &  E\left( p_i\left| w_{2i}; d_i=0\right. \right) =E\left( p_i\left| w_{2i}^0\right. \right) . \end{aligned}$$

Here, $$w_{2i}^1=(w_{2i},\widehat{p}_{3i})$$, $$w_{2i}^0=(w_{2i},1-\widehat{p}_{3i})$$ say $$w=(w_1,w_2)$$

Restrictions:

- Full Independence: $(\eta _1,\eta _2,\eta _3)$ and *w* are independent.
- Common support: for all $w_1 \in X$, the support of $(p_2^1, p_2^0, p_3)$ conditional on *w* and selection equals the support of $(p_1,p_2,p_3)$ conditional on selection.

In this model, if these assumptions are met, then $w_1$ and $\eta _1$ are independent conditional on $p_2^1$, $p_2^0$ and $p_3$ given selection. The approach amounts to estimating the conditional probability of selection and of the endogenous variable (propensity score) in the first step and in the second step approximating the correction function (control function) with a method such as polynomial series (Das et al., 2003) or pairwise differencing (our approach) in the second.

As it is well known, the identification constraint required in this framework is that at least one of the explanatory variables included in the participation equation and in the reduced equations be excluded from the outcome equation. To obtain estimators for the parameters of interest, assuming that the conditional distributions of $\eta _{1i}^1$ given ($w_i,\eta _{2i}^1,\eta _{3i}$) and $\eta _{1i}^0$ given ($w_i,\eta _{2i}^0,\eta _{3i}$) are parametric, it is possible to obtain estimators of the parameters of the work-absentee equation merely by straightforwardly calculating a Heckman’s two stage procedure (Kim, 2006). In this case, if the participation error is distributed as a standardized normal, it is appropriate to estimate a Probit model for the participation decision and also for the endogenous regressor equation. Unfortunately, if those strong assumptions do not hold, the resulting two stage estimators are asymptotically biased. To avoid this problem we include the unrestricted control functions and the components are estimated non-parametrically as discussed above. Thus, note that,

12 $$\begin{aligned} y_{i} = w_{1i}^{\top }\beta _1+d_i\lambda _1+ d_i\mu _1(p_{2i}^1,p_{3i})+(1-d_i)\mu _0(p_{2i}^0,p_{3i})+ u_{1i}, \end{aligned}$$

For the pairs (*i*, *j*) that fulfill

- $$\mu _1(p_{2i}^1,p_{3i}) = \mu _1(p_{2j}^1,p_{3j})$$, for $$i\ne j$$ and $$d_i=d_j$$,
- $$\mu _0(p_{2i}^0,p_{3i}) = \mu _0(p_{2j}^0,p_{3j})$$ for $$i\ne j$$ and $$d_i=d_j$$,

which results in

13 $$\begin{aligned} y_i - y_j= &  (w_{1i}-w_{1j})^{\top }\beta _1 + v_{1i} - v_{1j}, \quad i \ne j. \end{aligned}$$

We can proved that the least squares estimator of $\beta _1$ in (13) is consistent under (a) and (b) conditions. Thus, we used all pairs of observations and a weighted least squares estimator of $\beta _1$ giving greater weights to pairs for which $p_{2i}^1 \approx p_{2j}^1$, $p_{2i}^0 \approx p_{2j}^0$ and $p_{3i} \approx p_{3j}$, i.e.

14 $$\begin{aligned} \widetilde{\beta } = S^{-1}_{ww} S_{wy} \end{aligned}$$

where

15 $$\begin{aligned} S_{ww}= &  {N \atopwithdelims ()2}^{-1}\sum _i\sum _{i<j} \omega _{ij}(w_{1i}-w_{1j}) (w_{1i}-w_{1j})^{\top } \\ \nonumber S_{wy}= &  {N \atopwithdelims ()2}^{-1}\sum _i\sum _{i<j} \omega _{ij}(w_{1i}-w_{1j})(y_i-y_j). \end{aligned}$$

We are going to use the following weights

16 $$\begin{aligned} \nonumber \omega _{ij}= &  \frac{1}{h^2_2}K\left( \frac{p_{1i}-p_{1j}}{h}\right) p_ip_j d_id_j \\ &  + \frac{1}{h^2_2}K\left( \frac{p_{0i}-p_{0j}}{h}\right) p_ip_j (1-d_i)(1-d_j), \end{aligned}$$

where:

$$\begin{aligned} p_{1i}-p_{1j}= &  \left( p_{2i}^1-p_{2j}^1 \quad p_{3i}-p_{3j}\right) ^{\top } \\ p_{0i}-p_{0j}= &  \left( p_{2i}^0-p_{2j}^0 \quad p_{3i}-p_{3j}\right) ^{\top }, \end{aligned}$$

As $p_{2}^1$, $p_{2}^0$ and $p_{3}$ are unknown initially, it is necessary to use estimates of them to obtain a feasible estimator. Here, a non-parametric approach is proposed for estimating these propensities and then substituting them in the formula above to obtain the feasible estimator, $\hat{\beta _1}$.

## Footnotes

1 Note that $\beta _2$ and $\beta _3$ are not estimated since they are not identified in the semiparametric approach.

2 The whole process is shown in the Appendix.

3 The low-income threshold was defined based on the income intervals provided in the questionnaire. Specifically, individuals earning less than 970 euros per month were categorized as low income.Potential biases associated with self-reporting is mitigated using predefined income intervals.

4 According to the National Institute of Mental Health (1996), depression is the leading cause of absenteeism in the United States.

5 $Prob > F = 0.8228$, so the null hypothesis of parametric stability cannot be rejected.

6 Estimates are calculated using R and STATA statistics softwares.

7 ATE is generally understood as a causal parameter, i.e. an estimate or propensity of a population that a researcher wishes to know, defined without reference to the study design or estimation procedure. Both observational studies and the design of experimental studies with random assignment can enable one to estimate to ATEs in a variety of ways.
